# Humanitarian Corridors from War Zones for Vulnerable People and Those Under International Protection: An Example of Safe Migratory Flow Management in Italy

**DOI:** 10.3390/healthcare13131561

**Published:** 2025-06-30

**Authors:** Lavinia Bianco, Valerio Bianco, Giovanna Laurendi, Stefania Oliva, Mariarosaria Aromatario, Aline Pizzardi, Cristiano Camponi, Christian Napoli

**Affiliations:** 1 Department of Medical Surgical Sciences and Translational Medicine, “Sapienza” University of Rome, 00189 Rome, Italy; 2 National Institute for Health, Migration and Poverty (NIHMP), 00153 Rome, Italystefania.oliva@uniroma1.it (S.O.); mariarosaria.aromatario@inmp.it (M.A.); aline.pizzardi@inmp.it (A.P.);; 3 Department of Mathematics Guido Castelnuovo, “Sapienza” University of Rome, 00185 Rome, Italy; valeriobianco99@gmail.com; 4 Department of Public Health and Infectious Diseases, “Sapienza” University of Rome, 00185 Rome, Italy

**Keywords:** migration flow, humanitarian corridors, screening, PEPs

## Abstract

**Introduction**: Humanitarian Corridors are part of Protected Entry Procedures (PEPs), which allow for the safe and legal arrival in Europe of refugees in need of protection (art. 25 of Regulation (CE) n.810/2009) and were implemented for the first time in Italy in 2015. They represent an alternative to dangerous journeys, and they also bring benefits to local communities. The National Institute for Health, Migration and Poverty (NIHMP) represents the first filter at entry with regard to health needs, as it guarantees an overall individual health assessment to verify the possible presence of ongoing infectious pathologies and of issues that may require management and medical investigation. The aim of this study is to expose the organizational activity and analyze the sociodemographic and health data relating to the Humanitarian Corridors implemented in Italy and in which the NIHMP has participated from 2018 to 2024. **Materials and Methods**: The organizational lists and health data of each corridor were collected. The analysis was carried out in relation to trend, sociodemographic characteristics of the sample, identification, reception and health. The *p*-value was considered statistically significant if less than 0.01. In all cases in which the *p*-value was found to be statistically significant, Cramer’s V was calculated to evaluate the strength of the individual correlation. **Results**: The NIHMP has participated in 14 Humanitarian Corridors, allowing 1250 refugees to enter Italy; a total of 174 unaccompanied foreign minors (UFMs) arrived, and there were 451 refugees reported as vulnerable (36.1%). Contagious infectious conditions were identified in 223 refugees (17.8%), and other health findings were identified in 414 refugees (33.1%). In the analysis by sex, there are statistically significant differences in the reports of vulnerability and in nationality and education. The inferential analysis carried out by age groups presents statistically significant differences in the reports of vulnerability and in sex and nationality. The analysis relating to the differences by nationality shows statistically significant results in the individual corridors in the reports of vulnerability and in schooling, skin manifestations and infectious diseases and the presence of other health findings. **Conclusions**: The data reported and analyzed in this work can be considered the first attempt at a complete and detailed analysis regarding the actual implementation and effectiveness of Humanitarian Corridors as programs for legal and safe entry into Italy. The significant gap in research is the reason why there are characteristics of our sample that have no counterpart in the literature. Despite this, for other characteristics, it is possible to find statistical significance and scientific value in line with the data reported both on the websites dedicated to the topic and on the limited scientific literature on the subject.

## 1. Introduction

Despite the growing increase in both humanitarian needs and international migration (caused by increased economic disparities, energy crisis, socio-political instability and conflicts, increased population and climate change [1,2]), the humanitarian sector has often encountered difficulties and limitations; these limitations are linked to the poor coordination with local communities; to unpredictable, insufficient and difficult-to-administer funding; to the high level of aggression towards workers in this sector; to controversies and political battles; and also to the presence of multiple international organizations with the same aim [2,3]. Moreover, both public and academic research focuses more on the impact of the arriving populations on host societies rather than on the trauma suffered by refugees and migrants, with insufficient recognition of their health needs [4].

In order to encourage greater coordination between the organizations involved and to guarantee the safe and legal arrival in Europe of refugees in need of protection, “Protected Entry Procedures” (PEPs) have been created [5]. PEPs consist in the examination of the request for international protection at the Consulate of the destination Country located in a third Country (Country of origin or Country of temporary presence of the applicant), followed, in the event of a positive outcome, by the issuing of a visa; alternatively, a visa is directly issued for humanitarian needs to be examined in the destination Country [5].

For the implementation of the second option, in 2015, an experimental protocol for the creation of “Humanitarian Corridors” was signed by the competent authorities (Ministry of the Interior and the Ministry of Foreign Affairs) and the Community of Sant’Egidio, the Italian Federation of Evangelical Churches (FCEI), the Waldensian Table and the Italian Episcopal Conference [5,6,7,8]; this first protocol allowed, between 2016 and 2017, the safe arrival in Italy of 1011 refugees from Lebanon, who were welcomed according to the widespread reception model (integration of refugees in local realities) in 18 different Italian Regions [6]. As a result, Humanitarian Corridors, along with *resettlements*, “evacuations” happened during 2018, 2019, 2022 and 2024, and other pilot initiatives, such as University Corridors for students escaping from their own Countries, Health Corridors for minors affected by heart conditions [9] and Job Corridors [8,10], are one of the few for legal and safe entry into Europe [1].

### 1.1. Objectives of Humanitarian Corridors

Humanitarian Corridors were designed with various objectives, namely the following:

- To create a proven and replicable model offering vulnerable refugees safe passage and legal access to protection in Europe [7]: European Countries operate following Regulation (EC) no. 810/2009, which grants visas with limited territoriality “for humanitarian reasons or of national interest or by virtue of international obligations” in derogation from the principle of compliance with the entry conditions referred to in art. 5 of the Schengen Borders Code (Regulation (EU) 2016/399) [5,8,11,12].
- To reduce opportunities for human traffickers and smugglers to exploit vulnerable refugees [7]: Potential beneficiaries include displaced persons with a clear need for international protection, people identified as deserving of refugee status by the UNHCR (needs for legal and/or physical protection, survivors of torture and/or violence, medical needs, women and girls at risk, family reunification, children and adolescents at risk and lack of foreseeable lasting alternative solutions [7]) and individuals who, due to their vulnerability, would be easy victims of human trafficking or would have no possibility of reaching Europe, thus including the presence of serious pathologies and the condition of single mother [1].
- To ensure more effective integration into the local community [7]: In order to promote both integration into the social and productive fabric of the Country of destination and the achievement of autonomy, the beneficiaries are offered legal support for the request for international protection, healthcare and psychological support, cultural mediation and language courses, with school inclusion of minors, support courses for schooling outside school hours and active participation of parents in their children’s schooling, professional training and qualification courses, orientation to the world of work (activation of internships/work grants) and support in finding a housing solution [1,6,7].
- To achieve faster resolution of asylum claims: On average, a family unit needs a year and a half to reach full autonomy or, at least, significant semi-autonomy, while slightly shorter times are required for individuals, and feedback on the decisions on the asylum requests is given within six months, rather than two years as for refugees who enter Italy through other methods; this timing represents a significant success of this entry and reception model [6,7].
- To improve the general public’s perception of migrants (reduce negative preconceptions regarding immigration) [7].

### 1.2. Aim of the Study

The objective of this study is to analyze the sociodemographic and health data relating to the Humanitarian Corridors implemented in Italy and in which the Istituto Nazionale per la promozione della salute delle popolazioni Migranti e per il contrasto delle malattie della Povertà (INMP)—National Institute for Health, Migration and Poverty (NIHMP) participated from 2018 to today. NIHMP organization and management can be considered an Italian best practice, potentially susceptible to extension at the European level, also considering that the data relating to the actual implementation and effectiveness of the resettlement programs, often difficult to find and interpret, do not appear to have been analyzed in detail to date nor to have been the subject of publication in the scientific literature.

## 2. Background

In Italy, at the end of 2023, we reached the sixth protocol signed by various associations and with beneficiaries coming from different Countries, and the various Italian protocols allowed for the entry of a total of just under 10,000 refugees of various nationalities (Lebanon, Libya, Afghanistan, Ethiopia, Eritrea, Somalia, Sudan, Palestine, Iraq, etc.) [1].

Following the first Italian protocol, the model was exported to other European states, in particular France, Belgium, Andorra, Germany and Switzerland, albeit without continuity, with different programs and with more limited numbers [1,6,7,13]. Each Country decides autonomously how to manage local, national and international collaborations, as well as how to organize reception and integration tools [7], but even with this variability, the Humanitarian Corridors process lasts more than six months [7] and starts with various activities carried out in the Countries of transit, such as the collection of requests from the possible beneficiaries, with their identification and evaluation, before sending the list to the consular authorities of the Country of destination [6,7]. Furthermore, criminal record checks are carried out before allowing the start of bureaucratic procedures for issuing a humanitarian visa and establishing priority levels depending on the specific circumstances of each beneficiary, after which the flights are booked, and a health check is carried out [6,7]. A search for the final location is conducted based on the reception opportunities and the specific needs of each beneficiary (medical care, education opportunities, etc.), and activities to raise awareness among civil society on the topic and solidarity networks are activated throughout the Country of destination in order to identify those willing to support the reception and integration process of the beneficiaries into the local social fabric [6,7]. The beneficiaries are informed and trained regarding this humanitarian project; European and national legislation on political asylum and immigration; and the cultural, linguistic and socio-economic aspects of European culture, society and traditions [6,7].

### 2.1. Health Checks and Screenings

Once they arrive in the Country of destination, the beneficiaries are subjected to a new health check inside the airport and must sign the request for international protection at the Border Office before reaching their respective destinations [6,7]. However, even if a “screening” or a “health check” is always present upon arrival, some Countries provide an immediate check upon arrival, whereas others have two phases: health assessment on arrival followed by an integrated initiative after the granting of asylum [6,14]. Furthermore, through the use of dedicated questionnaires in many Countries, both European and from outside of Europe [12,15], it was possible to highlight the following:

- Both EU and non-EU Countries believe that screening migrants for diseases is useful [12,15];
- Many non-EU states are convinced that migrants can have an impact on the epidemiology of infectious diseases within their territory [15];
- Screening mainly concerns TB, HIV, HBV and HCV, with a higher prevalence of screening for sexually transmitted diseases in non-EU Countries [12,15].

As a general rule, healthcare reception in many European Countries has focused on screening for infectious diseases (tuberculosis, viral hepatitis and intestinal parasites), although it is important to also address the problem of non-communicable diseases (NCDs), including, due to the high percentage of affected refugees, mental health problems (affective and stress-related disorders) [14]. Indeed, chronic non-communicable diseases in refugees, although they vary widely across populations, include anemia, hypertension, impaired fasting glucose levels (e.g., diabetes), micronutrient deficiencies, chronic lung disease and overweight or obesity; furthermore, refugees have higher rates of chronic pain than the general population (65% to 83%), and their mental health and well-being are strongly influenced by their migration history, with a risk of developing post-traumatic stress disorder 10 times higher than the local population [16]. As for minors, in 2023, of the total number of forced migrants present in the world (117.3 million), 40% were minors [1,17], and the risk of disease in this population is influenced not only by political instability, war, poverty, poor hygiene and insufficient nutrition suffered in their Countries of origin but also by the lack of access to prenatal screening, vaccination programs and healthcare [18]; for this reason, the focus of the health assessment of refugee children should shift from the sole assessment of infectious diseases to an inclusive screening that allows for the early diagnosis of chronic and lifestyle-related conditions [18].

In Italy, in the rescue and initial reception phase, it is mandatory to carry out the following:

- Initial medical evaluation: the search for signs and symptoms indicative of clinical conditions requiring emergency/urgent healthcare;
- The detection of signs and/or symptoms: cough for ≥2 weeks (TB), fever, splenomegaly (malaria), skin inspection for ectoparasites, diarrhea, abdominal pain, nausea, vomiting, pruritus and signs and/or symptoms of anemia [19].

Furthermore, the medical examination must include the search for signs of trauma and/or results of torture, in consideration of the increasing presence of people with vulnerabilities within the incoming migratory flows and in order to guarantee the correct application of art. 17 of Legislative Decree no. 142/2015 [20], according to which reception measures must take into account specific vulnerabilities, such as “minors, unaccompanied minors, disabled people, the elderly, pregnant women, single parents with minor children, victims of human trafficking, people suffering from serious illnesses or mental disorders, people for whom it has been ascertained that they have suffered torture, rape or other serious forms of psychological, physical or sexual violence or violence linked to sexual orientation or the identity of gender, victims of female genital mutilation” [19,20,21]. In fact, the vulnerable have a series of guarantees and protection measures, including the issuing of dedicated certifications (e.g., victims of torture) useful for the recognition of international protection [21]. Finally, the use of cultural mediators in possession of specific healthcare backgrounds is recommended in order to support the doctor–patient relationship, as well as the use of information tools for the registration and ready availability of healthcare data throughout the reception process [19].

### 2.2. Role of NIHMP

These activities, starting in 2018, are carried out by the National Institute for Health, Migration and Poverty (NIHMP): by making available nurses, psychologists, transcultural mediators and various medical specialties (infectious disease specialists, internal medicine specialists, dermatologists and pediatricians), the NIHMP guarantees the carrying out of a complete individual health assessment, which allows for verifying the presence of ongoing infectious diseases transmissible via the respiratory and/or cutaneous route, with the simultaneous search for any signs, symptoms and/or vital parameters suggestive of pathologies that require management and medical investigation [22,23]. The presence of infectious pathologies precludes entry into community environments, while the identification of non-infectious issues upon arrival, with or without previously reported vulnerable cases, guarantees initial medical assistance and allows for referral to a specialist in deferrable cases and to healthcare facilities in the event of emergencies [22,23].

**Figure 1:**
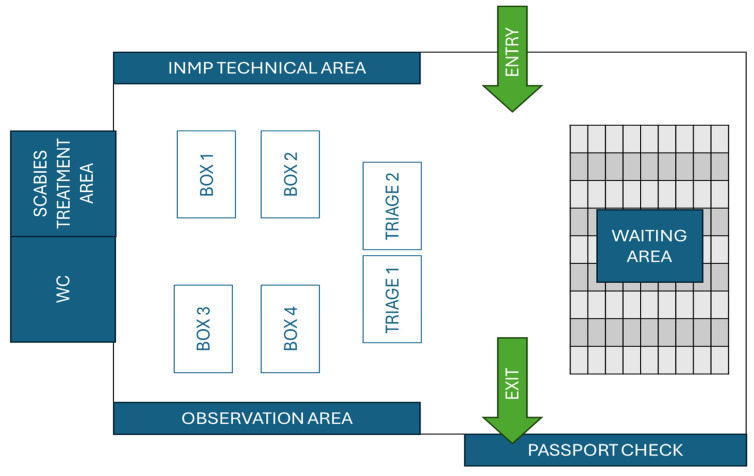
Example of a possible space distribution in the area destined to welcome refugees at Terminal 5 of Airport “Leonardo da Vinci” of Rome Fiumicino (Rome, Italy).

**Figure 2:**
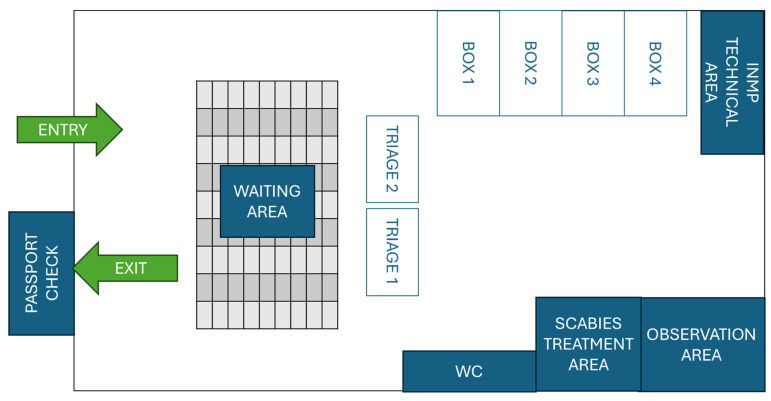
Example of a possible space distribution in the area destined to welcome refugees at Military Airport “M. De Bernardi” of Pratica di Mare-Pomezia (Rome, Italy).

NIHMP activities start by dividing the area identified for the disembarkation of passengers into a waiting area and a healthcare area. The healthcare area is composed of 1–2 nursing triage stations, in which the vital parameters are collected (pressure, temperature, heart rate and respiratory rate), 4–5 examination boxes, in which a medical examination with bodily inspection, percussion, palpation and auscultation, as well as possible immediate treatment (analgesic therapies), is carried out, a room for the treatment of scabies (usually a single application of permethrin 5% cream is sufficient to eliminate the infestation, but if the lesions do not heal or new ones appear, a second application can be done after 7 days [24]) and an observation area (Figure 1 and Figure 2). At the end of the medical examination, the NIHMP staff issues a certification relating to the presence or absence of any contagious pathologies and/or any clinical aspects that require further health investigations, a certification that allows for the continuation of the reception process; moreover, during the whole process, non-healthcare personnel support the prompt identification of vulnerable people through initial interviews with the help of linguistic–cultural mediators [21,22]. In case of scabies, treatment (application of permethrin 5% cream) is immediately administered, and the diagnosis is notified to both hosting communities and local sanitary agencies. If needed, it is possible to send refugees to the nearest emergency room via the vehicle made available by the 118 Operations Centre for the entire duration of the disembarkation procedures.

## 3. Materials and Methods

From 2018 to 2024, the NIHMP participated in 14 Humanitarian Corridors, which allowed 1250 refugees to enter Italy, and were distributed as such: 3 in 2018, 4 in 2019, 1 in 2021, 3 in 2022 and 3 in 2024.

The data reported in the registers relating to each corridor were collected and inserted into a spreadsheet. The data collected, which correspond to the registers’ headings, included the following:

- Corridor date;
- Sociodemographic characteristics: age, sex, nationality, marital status, level of education and occupation;
- Possible reporting of vulnerabilities by the UNHCR or other associations involved;
- The categories of refugees: family unit, single adult or unaccompanied foreign minor (UFM);
- The size and type of the family unit;
- Host Italian Region;
- The presence of signs, skin manifestations or symptoms of contagious infectious conditions;
- The possible presence of other data and/or health findings.

Data from all refugees arrived in the covered corridors were included, as there were no exclusion criteria. All data present in the registries were collected by two researchers and double-checked by a third one before starting the analysis. Due to the nature of our data, there were not any missing data, as the dataset was based on the obligatory sections of the registers, and therefore, there could not be any empty data; any year with a number of refugees equal to zero is due to the fact that there were no Humanitarian Corridors in that year.

The analysis of the sample was carried out in relation to the trend, sociodemographic characteristics, identification and reception. Finally, health data were analyzed. R software, version 4.4.0 (2024-04-24 UCRT), was used; the spreadsheet did not need to undergo further modifications or optimizations before being imported into R.

Regarding the descriptive analysis, the continuous variables such as the trend were expressed as the median and first and third quartiles, except for the variable age, which was also expressed as the mean and standard deviation. Categorical variables such as nationality were summarized as the number and percentage of refugees for each category.

Inferential statistical analysis was carried out considering gender, age and nationality and comparing them with the other usable variables. To perform the inferential statistical analysis and, therefore, the comparisons between the various data available to us, the Chi Square Test was used, and a *p*-value was considered statistically significant if less than 0.01. In all cases in which the *p*-value was found to be statistically significant, Cramer’s V (a measure of the effect size for the Chi Square independence test) was calculated to evaluate the strength of the individual correlation, so if the effect size (ES) is ≤0.2 the correlation is weak; if it is 0.2 < ES ≤ 0.6, the correlation is moderate; and if ES > 0.6, the correlation is strong [25].

In order to allow for the analysis, some of the data collected were merged to erase the zeros: for example, the nationalities of Cameroon, Chad, Congo, Egypt, Ethiopia, Myanmar, Nigeria, the Syrian Arab Republic, Somalia and Yemen have been grouped as “Other”, as they have fewer than 5 refugees or are not even represented in the majority of corridors. For the diagnoses of skin manifestations and/or infectious diseases, only scabies and tuberculosis (TB) were considered separately from the rest, as they are the most relevant pathologies within the sample. In the inferential statistical analysis divided by sex, the “Woman at Risk” vulnerability was excluded in its entirety, as it was present only in women, while in the one that included the comparison between the individual corridors, corridors no. 8 (25 November 2021) and no. 14 (2 September 2024) were excluded, as the number of refugees on the two dates was less than 5.

Regarding the statistical analysis by age groups, three age groups were used: under 18 years, between 19 and 30 years and over 30 years. The groups were created using quartiles as an indicator in conjunction with the desire to separate minors from the rest. It was not possible to make a comparison with the diagnoses of skin manifestations and/or infectious diseases, as the number of people affected by TB in the 19–30 age group was less than 5. Likewise, a number of less than 5 did not allow for an analysis relating to the reception sites, not even considering the macro-areas (northern, central, southern and insular).

In the mentioned cases of fewer than 5, it was not possible to replace the Chi Square Test with Fisher’s Exact Test, as the rest of the sample was too large to apply this test and obtain reliable results.

## 4. Results

**Figure 3:**
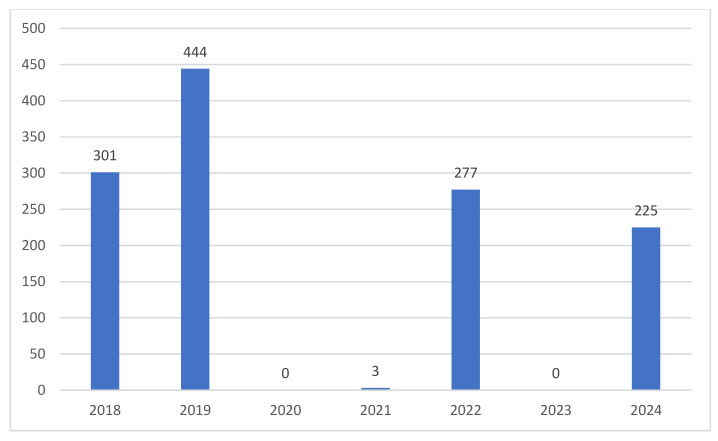
Yearly distribution of refugees.

The distribution of refugees over the years from 2018 to 2024, as well as for each corridor, does not appear to be homogeneous, considering that, in the various years, the minimum number of landings is 3, the maximum number is 444, and the median is equal to 225 (Q1 1.5 and Q3 289) (Figure 3).

**Figure 4:**
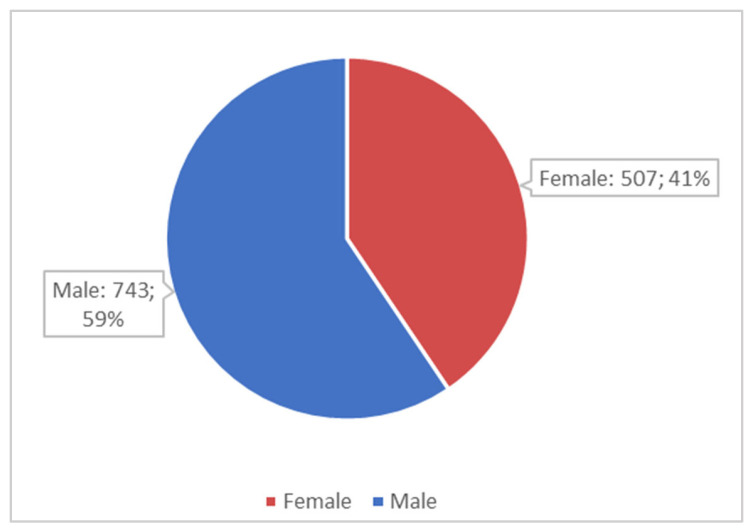
Gender distribution.

With regard to sociodemographic characteristics, it was found that during the 7 years analyzed, 743 male and 507 female refugees arrived in Italy, equal to 59.4% and 40.6% of the sample, respectively (Figure 4). In total, 326 minors arrived (26.1% of the total arrivals), as well as 924 adults (73.9% of the total arrivals).

**Figure 5:**
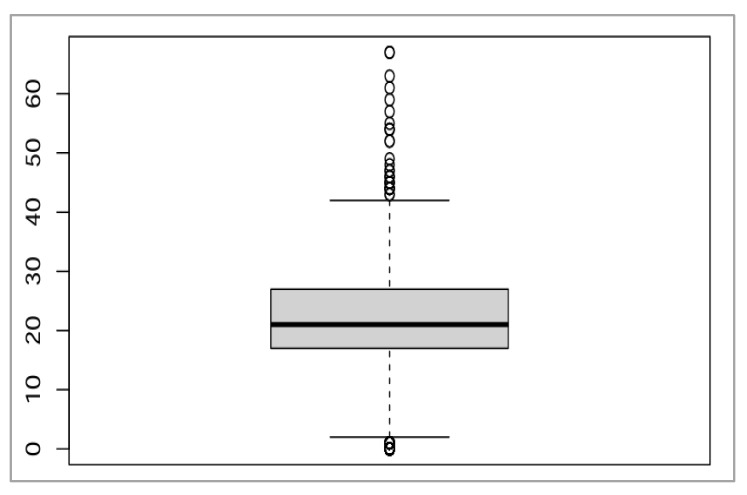
Age boxplot.

The mean age was 21.7 ± 9.21, with a range of 0–67, while the median was 21 (Q1 17 and Q3 27) (Figure 5).

**Figure 6:**
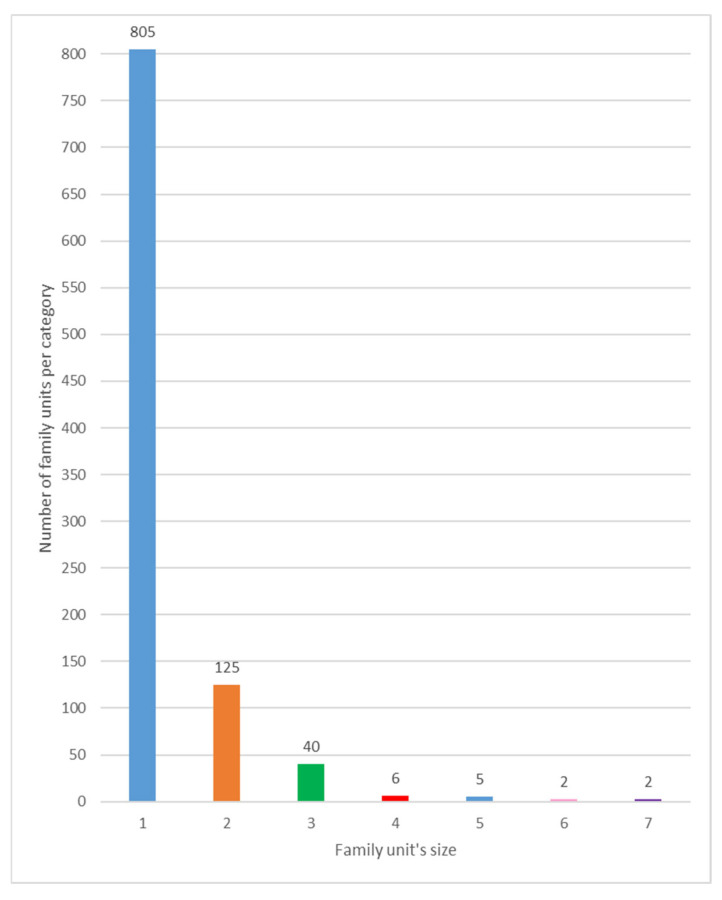
Distribution of family units per number of components.

Refugees were categorized into family units, single adults or unaccompanied foreign minors (UFMs); both single adults and UFMs were classified as a family unit by a single expert, so family units ranged from a minimum of 1 to a maximum of 7 members (Figure 6); most refugees arrived alone (805 family units of 1), while large family units (6 or 7 members) were found to be only 4, for a total of 26 refugees.

A total of 174 UFMs arrived along the various corridors (13.9% of the total sample), most of whom were 16 years old (68, equal to 39.1% of all UFMs); the other most represented ages were 17 years old (44, 25.3% of all UFMs) and 15 years old (36, 20.7% of all UFMs), but the two youngest minors (1.1% of all UFMs) declared they were 12 years old.

The most represented nationalities were Eritrean (44.8%), Sudanese (26.6%), Somalian (16.0%) and Ethiopian (8.3%) (Table 1), while in relation to education and employment, in most cases, it was not possible to find the information (respectively, 811 refugees and 902 refugees, equal to 64.9% and 72.2% of the sample). The marital status as well was unknown in 649 cases (51.9% of the total sample).

There were 451 reports of vulnerability, meaning there was a report in 36.1% of the sample; the most frequent warning was “Detained/Held” (33.3% of the total reports), followed by “Health problems/Malnutrition/Chronic pathology” (17.5%), “Woman at Risk” (14.2%) and “Child at Risk” (13.7%). The year with the highest number of annotations was 2019, in which 255 refugees arrived in Italy with a vulnerability report, while the year with the lowest number was 2021, with only 3 refugees reported, all highlighted as “Child at Risk” (Table 2).

**Figure 7:**
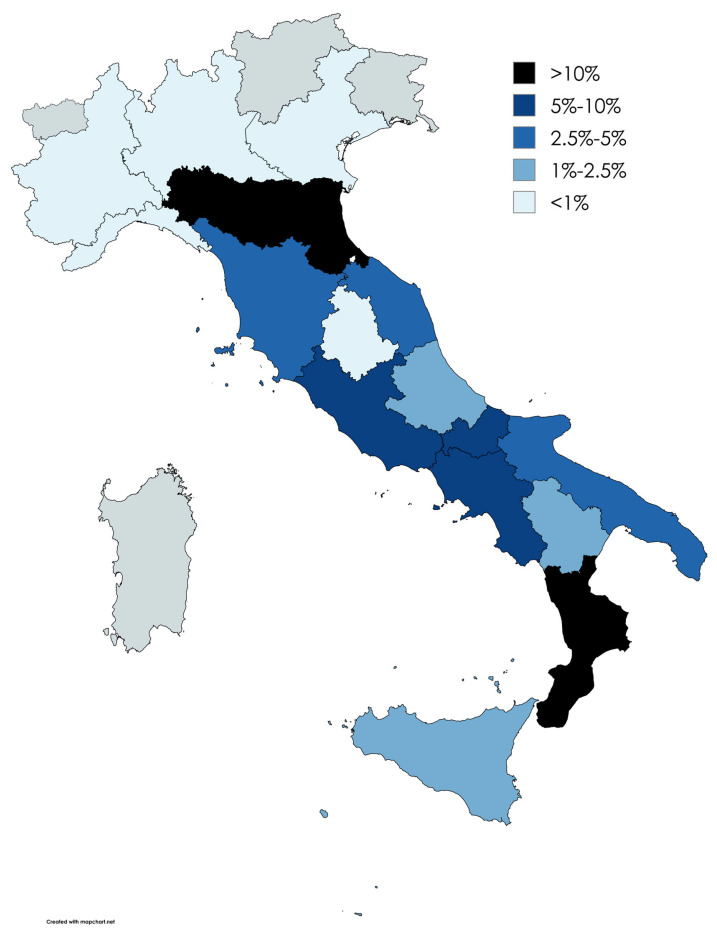
Choropleth map of the host Italian Regions.

Of the 1250 refugees welcomed between 2018 and 2024, 825 (66.0%) were distributed among 16 Italian Regions; the three most represented Regions are Emilia-Romagna (189 refugees), Calabria (153 refugees) and Lazio (86 refugees) (Table 3 and Figure 7).

Signs, skin manifestations or symptoms of contagious infectious conditions were reported in 223 refugees (17.8% of the total sample); the most commonly diagnosed disease was scabies (58 cases, equal to 26.0% of the total refugees affected by contagious infectious conditions), followed by cough lasting more than 5 days (29 cases, 13.0%), tuberculosis (27 cases, 12.1%), acute nonspecific symptoms (26 cases, 11.6%), fungal infection (25 cases, 11.2%) and parainfluenza symptoms (24 cases, 10.7%) (Table 4).

Other health findings were diagnosed and/or identified in 414 refugees (33.1% of the total sample), of whom 89 (7.1% of the total sample) were under 18 years of age. This category includes all other possible health information, from the presence of scars to myopia to headache to tooth decay, but also includes chronic non-communicable diseases, for a total of 53 refugees (4.2%) affected and distributed as follows:

- 18 refugees (1.4%) with hypertension, all adults;
- 10 (0.8%) with diabetes, all adults;
- 9 (0.7%) with psychological/psychiatric problems, of which 2 were minors;
- 7 (0.6%) with cardiac rhythm abnormalities, of which 1 was a minor;
- 4 (0.3%) with heart disease, all under the age of 18;
- 3 (0.2%) with asthma, of which 1 was a minor;
- 1 (0.1%) in adulthood with anemia and 1 (0.1%) minor with epilepsy.

The results of the inferential statistical analysis by gender are reported in Table 5. One of the statistically significant differences is related to reports of vulnerability (*p* < 0.01) and can be seen from Cramer’s V, which shows a moderate correlation (0.2 < ES ≤ 0.6). Another significant *p*-value is found in relation to education (*p* < 0.01), but in this case the correlation is weak (ES ≤ 0.2), and in relation to nationality (*p* < 0.01), with a moderate correlation (0.2 < ES ≤ 0.6).

However, there is no statistically significant difference in relation to health data.

The inferential analysis carried out by age group is reported in Table 6. Three age groups were used—less than or equal to 18 years, between 19 and 30 years and greater than or equal to 31 years—and the table shows that the majority of the sample falls within the 19–30 age group.

The reports of vulnerability present statistically significant differences (*p* < 0.01), and this can be seen from Cramer’s V, which shows a moderate correlation (0.2 < ES ≤ 0.6). Further significant *p*-values are found in relation to gender and nationality, with a *p*-value less than 0.01 and weak correlation (ES ≤ 0.2) in both cases.

The statistical analysis of differences by nationality is presented in Table 7. For the individual corridors, a *p*-value lower than 0.01 and a Cramer’s V between 0.4 and 0.6 are found, indicating a statistically significant difference with moderate correlation.

The differences present in the sample regarding reports of vulnerability, education, skin and infectious disease manifestations and the presence of other health findings are also statistically significant (*p* < 0.01) but with a weak correlation (ES ≤ 0.2).

The results of the inferential statistical analysis by type of vulnerability and reported health data are reported in Table 8.

The only statistically significant difference concerns the presence of other health findings (*p* < 0.01), but the strength of the correlation obtained with Cramer’s V is weak (ES ≤ 0.2).

The results of the inferential statistical analysis by level of education and reported health data are reported in Table 9.

There are no statistically significant differences in regard to the presence of health findings and level of education.

## 5. Discussion

The functioning and organization of humanitarian supply networks are the primary topics of the current scientific literature [26,27,28,29,30], so it is challenging to assess the congruence of NIHMP data with those presently available. Furthermore, since humanitarian situations are frequently brought on by natural disasters or conflicts, their dynamic context rarely allows for the collection of comprehensive and reliable quantitative information [30,31].

Looking at the 3632 refugees welcomed into Italy from February 2016 to January 2022, the Community of Sant’Egidio reports that the most represented Countries of origin were Lebanon (2151 refugees, 59.2%) and Ethiopia (817 refugees, 22.5%), followed by Greece, Niger, Afghanistan, Libya, Jordan, Cyprus and Turkey [13]. As of 2023, the UNHCR reports that 73% of all refugees are from Afghanistan (6.4 million), the Syrian Arab Republic (6.4 million), Venezuela (6.1 million), Ukraine (6.0 million) and South Sudan (2.3 million) [1,17]. In the NIHMP sample, this figure is not confirmed, since the most represented Countries of origin are Eritrea (44.8%) and Sudan (26.6%). According to reports, in 2023, there was a spike in forced migration from Sudan with the onset of a conflict [1], which is consistent with our data; the spike began in the second half of 2022, most likely as a result of attempts to flee during the early stages of the conflict. The same trend has been reported regarding the Democratic Republic of the Congo and Myanmar [1]; however, it is not reflected in our data. In absolute terms, Syria is the Country most affected by forced migration [1], especially since 2016, when the war led to the largest wave of refugees in decades [32], but it is one of the least represented Countries of origin in our sample, with the highest number of refugees per corridor being 10; nevertheless, it is indisputable that Syria is more prevalent in the 2022 Humanitarian Corridors. Lastly, the fact that 92% of Somalia’s arrivals in our sample occurred in 2018 and 2019 may be related to the Country’s severe drought, which started in 2015, peaked at the start of 2017, and led to the registration of 167,000 displaced Somali refugees in 2018 [33].

The number of forced migrants in the world increased almost sixfold between 2000 and 2023, from 20 million (counted since 1951) to 117.3 million, of whom approximately 47 million (40%) are children under the age of 18, despite the fact that minors make up only 30% of the world’s population [1,17]. This high number of minors is also consistent with the data from the Humanitarian Corridors followed by the NIHMP: in fact, unaccompanied minors were equal to 13.9% of the total arrivals, while accompanied foreign minors were 26.1% (in 2019, minors were equal to 47.1% of the total arrivals in the same year, and in 2021, the refugees who arrived were all unaccompanied minors). However, during 2023, there was an irregular pattern of entry into Europe, with minors equal to only 10% of the total number of entries [1], and in our sample, it is possible to see that most of the corridors from 2022 to date have a reduced number of minors: specifically, in 2022, they were 5.1% of the total arrivals and, in 2024, 10.2% of the total arrivals.

With regard to gender, focusing only on what was recorded during 2023, 10% of irregular migrations to Europe were carried out by women [1]. This is not consistent with our numbers, as between the year 2022 and the year 2024, female refugees accounted for about 30% of arrivals, probably because the Humanitarian Corridors are managed by various organizations whose purpose is to ensure safe border crossing for the most vulnerable refugees, such as women.

Most Western European Countries screen migrants for active tuberculosis on arrival or shortly thereafter, regardless of the incidence of TB in the migrants’ Countries of origin, and the same can be said for HIV, HBV and HCV [14,34]. However, it is important to remember that migrants’ health outcomes depend on the conditions and mode of travel, including the number and type of potentially traumatic experiences [35,36]; for example, sexually transmitted diseases are closely linked to sexual violence, which can occur during transit and displacement in unsafe transit Countries [35]. As a result, it is possible that the reduced number of diagnoses of cutaneous and/or respiratory infectious diseases identified in our sample during the health screening upon arrival (17.8% of the total sample) is due to the fact that refugees arriving through Humanitarian Corridors are subject to greater health protections both before departure and during transit. In any case, a common finding in the literature is that migrants do not pose a significant risk to the populations of the European Union in increasing the incidence of infectious diseases and in the development of outbreaks [37].

Chronic non-communicable diseases identified as high risk in the migrant population (cardiovascular, musculoskeletal and respiratory diseases) are less studied, although they may represent an additional burden on the destination Countries’ health systems [35]. For this reason, and to ensure timely management by the Italian National Health Service (SSN—Servizio Sanitario Nazionale), during health screening, the NIHMP collects additional information on signs, symptoms and previous diagnoses of chronic non-communicable diseases. In our sample, these illnesses are a limited number (4.2% of the total sample), but their distribution is consistent with what has been reported in the literature, according to which chronic non-communicable diseases in refugees include, among others, anemia, hypertension, altered fasting glucose levels (e.g., diabetes), chronic pain and problems related to their mental health [16]. The same statement cannot be made with the pediatric population, as the epidemiology of refugee children recognizes high prevalence rates, in order, of vitamin D deficiency, intestinal infections, anemia, latent tuberculosis infection, hemoglobinopathies and chronic HBV upon entry into the host Countries [18], while in our sample, heart diseases are the most represented, followed by psychological/psychiatric problems.

With regard to reception, migrants depend on non-profit organizations, private networks and voluntary associations, which, through direct support, capacity building and advocacy, and also thanks to the government’s support or financing, allow migrants to build an identity, address the negative conceptions often present towards them in the Country of arrival, overcome barriers and acclimatize to society [38]; however, the scientific literature does not have a homogeneous collections of data on reception places in Italy, so it is particularly challenging to assess the consistency of our sample with what happens outside the Humanitarian Corridors. This difficulty is also enhanced by the high number of refugees that arrived through Humanitarian Corridors for whom it was not possible to know the host Province. Despite this, it is possible to highlight that the three most represented Italian Regions are Emilia-Romagna, Calabria and Lazio; it is interesting to note that, looking at the distribution by Provinces, these do not entirely correspond to the distribution by Region: the three Provinces that have received the largest number of refugees are Reggio Calabria (Calabria Region), Campobasso (Molise Region) and Rome (Lazio Region). In the distribution by Regions, the case of Emilia-Romagna is consistent with the data relating to the SAI network, as it represents the first Region in northern Italy for the number of active places (3506) [1]. The presence of Calabria and Lazio in the top positions is also in line with the available data, as Calabria has 115 SAI projects for 3238 active places, and Lazio has 41 for 2587 places; instead, it is surprising that Lombardy is not well represented in our sample (only seven refugees were welcomed into this Region), being in the first place for the number of projects and having more than 3000 active places [1].

## 6. Conclusions

The data reported and analyzed in this work can be considered the first attempt at a complete and detailed analysis of the effectiveness of Humanitarian Corridors as a legal and safe entry program in Italy.

The lack of scientific articles focusing on Humanitarian Corridors in favor of articles focusing on humanitarian supply chains suggests a significant gap in the research, which is why there are characteristics specific to our sample, such as reports of vulnerability and host locations, that have applicability and statistical significance only within our internal analysis, as they have no counterparts in the literature.

Despite this, it is possible to find statistical significance and scientific value with regard to the progressive increase in forced migration from Sudan, the significant incidence of underage migrants and the distribution of chronic non-communicable diseases among adult refugees, elements that are in line with the data reported in the scarce scientific literature on the subject; moreover, it is possible to find statistical significance and scientific value with regard to the different incidences in the populations of Humanitarian Corridors compared to those of migrants who arrive irregularly in relation to the high presence of migrant women, the reduced incidence of diagnoses of cutaneous and/or respiratory infectious diseases in our sample and the different distributions of chronic non-communicable diseases among underage refugees.

Finally, the statistical significance found relating to the reports of vulnerability by sex, by age and by nationality appears particularly interesting, although without a counterpart in the literature; it would be useful to carry out further investigations in other types of PEPs, also in relation to the different distributions of statistical significance found in the generic “other health findings” in the various categories of vulnerabilities identified by the UNHCR.

This study has some limitations. First, considering the gap in the current scientific literature, it is challenging to assess the congruence of NIHMP data with those presently available; secondly, as the reported data and the medical documentation data were taken from anonymous databases, it was not possible to recover missing data by asking the refugees directly. Lastly, only data from Humanitarian Corridors managed by the NIHMP was included, excluding any other corridors that may have happened in the same period but were managed by some other association.

## Figures and Tables

**Table 1:** Nationalities present in the sample.

Country of Origin	2018	2019	2021	2022	2024	Number of Refugees	Percentage of the Sample Total
Cameroon	1	0	0	3	3	7	0.56%
Chad	0	0	0	2	0	2	0.16%
Democratic Republic of the Congo (DRC)	5	0	1	1	0	7	0.56%
Egypt	0	0	0	0	2	2	0.16%
Eritrea	141	260	0	82	77	560	44.80%
Ethiopia	33	19	0	31	21	104	8.32%
Myanmar	0	0	0	0	2	2	0.16%
Nigeria	0	0	0	4	0	4	0.32%
Somalia	76	108	2	5	9	200	16.00%
Sudan	43	52	0	128	110	333	26.64%
Syrian Arab Republic	0	5	0	19	1	25	2.00%
Yemen	0	0	0	2	0	2	0.16%
Unknown	2	0	0	0	0	2	0.16%
Total	301	444	3	277	225	1250	100.00%

**Table 2:** Distribution of the reports of vulnerability.

	2018	2019	2021	2022	2024	Number of Refugees	Percentage fn the Sample Total
Child at risk	26	15	3	2	16	62	13.75%
Detained/held	0	150	0	0	0	150	33.26%
Detained/held—victim of torture and/or sexual/physical violence	0	12	0	0	0	12	2.66%
Possible or certain pregnancy	6	13	0	2	1	22	4.88%
LGBTIQ	0	1	0	1	3	5	1.11%
Health problems/malnutrition/chronic pathology	5	28	0	24	22	79	17.52%
Specific legal, economic and physical protection needs	13	7	0	0	0	20	4.43%
Victim of torture and/or physical violence	0	13	0	1	6	20	4.43%
Victim of gender-based and/or sexual violence	4	9	0	0	4	17	3.77%
Woman at risk	50	7	0	7	0	64	14.19%
Total	104	255	3	37	52	451	100.00%

**Table 3:** Distribution of the sample in the host Italian Regions.

	2018	2019	2021	2022	2024	Number of Refugees	Percentage of the Sample Total
Abruzzo	0	7	0	6	7	20	1.60%
Basilicata	15	0	0	8	2	25	2.00%
Calabria	0	0	0	48	105	153	12.24%
Campania	0	25	0	49	0	74	5.92%
Emilia-Romagna	17	165	0	4	3	189	15.12%
Lazio	29	38	0	6	13	86	6.88%
Liguria	0	0	0	0	7	7	0.56%
Lombardia	0	0	0	0	7	7	0.56%
Marche	8	27	0	3	19	57	4.56%
Molise	0	52	0	1	22	75	6.00%
Piemonte	0	0	0	6	4	10	0.80%
Puglia	0	7	0	13	33	53	4.24%
Sicilia	0	0	0	13	0	13	1.04%
Toscana	34	2	0	10	0	46	3.68%
Umbria	0	0	0	0	3	3	0.24%
Veneto	0	0	0	7	0	7	0.56%
Unknown	198	121	3	103	0	425	34.00%
Total	301	444	3	277	225	1250	100.00%

**Table 4:** Signs, skin manifestations or symptoms of contagious infectious conditions reported by NIHMP health personnel.

	2018	2019	2021	2022	2024	Number of Refugees	Percentage of the Sample Total
Acariasis	2	1	0	0	0	3	1.35%
Dysentery	1	1	0	0	0	2	0.90%
Viral hepatitis	1	1	0	2	4	8	3.59%
HIV+	0	0	0	1	0	1	0.45%
Fungal infection	8	5	1	2	9	25	11.21%
Suspected or confirmed upper respiratory tract infection	2	1	0	0	0	3	1.35%
Viral infection	0	0	0	2	1	3	1.35%
Hansen disease	0	0	0	0	1	1	0.45%
Multiple contagious infectious conditions	0	4	0	0	0	4	1.79%
Intestinal parasitosis	0	0	0	1	0	1	0.45%
Pneumonia	1	0	0	0	0	1	0.45%
Possible STD	2	1	0	0	1	4	1.79%
Scabies	16	22	1	12	7	58	26.01%
Acute nonspecific symptoms	11	8	0	1	6	26	11.66%
Parainfluenza symptoms	10	13	0	0	1	24	10.76%
Suspected exanthematous disease	2	1	0	0	0	3	1.35%
TBC	1	18	0	4	4	27	12.11%
Cough lasting more than 5 days	17	10	0	1	1	29	13.00%
Total	74	86	2	26	35	223	100.00%

**Table 5:** Results of inferential analysis of females vs. males.

	All	Females	Males	*p*-Value *	Cramer’s V
n	1250	507	743	-	-
Age (years), mean (SD, min–max)	21.7 ± 9.21 (0–67)	20.8 ± 9.1(0–67)	22.3 ± 9.9(0–67)	-	-
Reports of vulnerability ^ç^				**0.00006209**	*0.3132742*
None, n (%)	799 (63.9%)	293 (57.8%)	506 (68.1%)		
Detained/held, n (%)	150 (12.0%)	53 (10.5%)	97 (13.0%)		
Health problems, n (%)	79 (6.3%)	19 (3.7%)	60 (8.1%)		
Child at risk, n (%)	62 (5.0%)	22 (4.3%)	40 (5.4%)		
Other, n (%)	96 (7.7%)	56 (11.0%)	40 (5.4%)		
Signs, skin manifestations or symptoms of contagious infectious conditions				0.03525133	-
None, n (%)	1027 (82.2%)	425 (83.8%)	602 (81.0%)		
Scabies, n (%)	58 (4.6%)	16 (3.2%)	42 (5.7%)		
TBC, n (%)	27 (2.2%)	6 (1.2%)	21 (2.8%)		
Other, n (%)	138 (11.0%)	60 (11.8%)	78 (10.5%)		
Other health findings				0.4945061	**-**
None, n (%)	836 (66.9%)	333 (65.7%)	503 (67.7%)		
Present, n (%)	414 (33.1%)	174 (34.3%)	240 (32.3%)		
Education				**0.00000203**	0.1596729
None or informal, n (%)	216 (17.3%)	107 (21.1%)	109 (14.7%)		
Between 1 and 5 years, n (%)	61 (4.9%)	14 (2.8%)	47 (6.3%)		
Between 6 and 8 years, n (%)	90 (7.2%)	18 (3.5%)	72 (9.7%)		
Equal or higher than 9 years, n (%)	72 (5.8%)	27 (5.3%)	45 (6.1%)		
Unknown, n (%)	811 (64.9%)	341 (67.3%)	470 (63.2%)		
Nationality				**<0.0000001**	*0.2623697*
Eritrean, n (%)	560 (44.8%)	249 (%)	311 (%)		
Sudanese, n (%)	333 (26.6%)	67 (%)	266 (%)		
Other, n (%)	357 (28.6%)	191 (%)	166 (%)		

* Chi Square Test. ç Category “Woman at Risk” was excluded: selection bias (64 women, 5.1% of the sample). **Bold:** *p*-value less than 0.01. *Italics:* Cramer’s V is 0.2 < ES ≤ 0.6 (the correlation is moderate).

**Table 6:** Results of inferential analysis by age group.

	All	Less Than or Equal to 18 Years	Between 19 and 30 Years	Greater Than or Equal to 31 Years	*p*-Value *	Cramer’s V
n	1250	404	677	169	-	-
Age (years), mean (SD, min–max)	21.7 ± 9.21 (0–67)	12.0 ± 6.6 (0–18)	23.6 ± 3.2 (19–30)	37.4 ± 7.3 (31–67)	-	-
Reports of vulnerability					**<0.0000001**	*0.2773027*
None, n (%)	799 (63.9%)	236 (58.4%)	440 (65.0%)	123 (72.8%)		
Detained/held, n (%)	150 (12.0%)	68 (16.8%)	74 (11.0%)	8 (4.7%)		
Health problems, n (%)	79 (6.3%)	15 (3.7%)	47 (7.0%)	17 (10.1%)		
Child at risk, n (%)	62 (5.0%)	62 (15.4%)	0 (0.0%)	0 (0.0%)		
Woman at risk, n (%)	64 (5.1%)	4 (1.0%)	55 (8.0%)	5 (3.0%)		
Other, n (%)	96 (7.7%)	19 (4.7%)	61 (9.0%)	16 (9.4%)		
Gender					**0.0000002**	0.1561605
Females, n (%)	507 (40.6%)	134 (33.2%)	322 (47.6%)	51 (30.2%)		
Males, n (%)	743 (59.4%)	270 (66.8%)	355 (52.4%)	118 (69.8%)		
Nationality					**0.0001204**	0.09614463
Eritrean, n (%)	560 (44.8%)	195 (48.3%)	305 (45.1%)	60 (35.5%)		
Sudanese, n (%)	333 (26.6%)	97 (24.0%)	166 (24.5%)	70 (41.4%)		
Other, n (%)	357 (28.6%)	112 (27.7%)	206 (30.4%)	39 (23.1%)		
Signs, skin manifestations or symptoms of contagious infectious conditions					0.2019076	-
None, n (%)	1027 (82.2%)	322 (79.7%)	568 (83.9%)	137 (81.1%)		
Present, n (%)	223 (17.8%)	82 (20.3%)	109 (16.1%)	32 (18.9%)		

* Chi Square Test. **Bold:** *p*-value less than 0.01. *Italics:* Cramer’s V is 0.2 < ES ≤ 0.6 (the correlation is moderate).

**Table 7:** Results of inferential analysis by nationality.

	All	Eritrea	Sudan	Other	*p*-Value *	Cramer’s V
n	1250	560	333	357	**-**	**-**
Age (years), mean (SD, min–max)	21.7 ± 9.21 (0–67)	20.9 ± 8.9 (0–61)	23.6 ± 11.1 (0–67)	21.1 ± 8.9 (0–63)	**-**	**-**
Humanitarian Corridors ^@^					**<0.0000001**	*0.4169473*
I corridor, n (%)	148 (11.8%)	97 (17.3%)	2 (0.6%)	49 (13.7%)		
II corridor, n (%)	50 (4.0%)	6 (1.1%)	30 (9.0%)	14 (3.9%)		
III corridor, n (%)	103 (8.3%)	38 (6.8%)	11 (3.3%)	54 (15.1%)		
IV corridor, n (%)	144 (11.6%)	70 (12.5%)	7 (2.1%)	67 (18.8%)		
V corridor, n (%)	148 (11.8%)	126 (22.5%)	7 (2.1%)	15 (4.2%)		
VI corridor, n (%)	98 (7.8%)	55 (9.8%)	16 (4.8%)	27 (7.6%)		
VII corridor, n (%)	54 (4.3%)	9 (1.6%)	22 (6.6%)	23 (6.4%)		
IX corridor, n (%)	97 (7.8%)	46 (8.2%)	33 (9.9%)	18 (4.9%)		
X corridor, n (%)	79 (6.3%)	19 (3.4%)	43 (12.9%)	17 (4.8%)		
XI corridor, n (%)	101 (8.0%)	17 (3.0%)	52 (15.6%)	32 (9.0%)		
XII corridor, n (%)	119 (9.5%)	32 (5.7%)	70 (21.0%)	17 (4.8%)		
XIII corridor, n (%)	102 (8.2%)	45 (8.1%)	40 (12.1%)	17 (4.8%)		
Reports of vulnerability					**<0.0000001**	0.1733129
None, n (%)	799 (63.9%)	343 (61.3%)	242 (72.7%)	214 (59.9%)		
Detained/held, n (%)	150 (12.0%)	99 (17.7%)	22 (6.6%)	29 (8.1%)		
Health problems, n (%)	79 (6.3%)	26 (4.6%)	27 (8.1%)	26 (7.3%)		
Child at risk, n (%)	62 (5.0%)	22 (3.9%)	23 (6.9%)	17 (4.8%)		
Woman at risk, n (%)	64 (5.1%)	34 (6.1%)	1 (0.3%)	29 (8.1%)		
Other, n (%)	96 (7.7%)	36 (6.4%)	18 (5.4%)	42 (11.8%)		
Education					**<0.0000001**	0.1560971
None or informal, n (%)	216 (17.3%)	63 (11.3%)	4 (14.7%)	104 (29.1%)		
Between 1 and 5 years, n (%)	61 (4.9%)	24 (4.3%)	14 (4.2%)	23 (6.4%)		
Between 6 and 8 years, n (%)	90 (7.2%)	33 (5.9%)	22 (6.6%)	17 (4.8%)		
Equal or higher than 9 years, n (%)	72 (5.8%)	53 (9.5%)	21 (6.3%)	16 (4.5%)		
Unknown, n (%)	811 (64.9%)	387 (69.0%)	227 (68.2%)	197 (55.2%)		
Signs, skin manifestations or symptoms of contagious infectious conditions					**0.00730261**	0.083913658
None, n (%)	1.027 (82.2%)	447 (79.8%)	293 (88.0%)	287 (80.4%)		
Scabies, n (%)	58 (4.6%)	36 (6.4%)	6 (1.8%)	14 (3.9%)		
TBC, n (%)	27 (2.2%)	14 (2.5%)	3 (0.9%)	10 (2.8%)		
Other, n (%)	138 (11.0%)	63 (11.3%)	31 (9.3%)	46 (12.9%)		
Other health findings					**0.008737384**	0.087087491
None, n (%)	836 (66.9%)	400 (71.4%)	210 (63.1%)	226 (63.3%)		
Present, n (%)	414 (33.1%)	160 (28.6%)	123 (36.9%)	131 (36.7%)		

* Chi Square Test. @ Excluded corridors n. VIII (25 November 2021) and n. XIV (2 September 2024): numerosity less than 5, with a total of 7 refugees (0.6% of the sample), whose nationalities are in the category “Other”. **Bold:** *p*-value less than 0.01. *Italics:* Cramer’s V is 0.2 < ES ≤ 0.6 (the correlation is moderate).

**Table 8:** Results of inferential analysis by type of vulnerability reported.

	Sample with Vulnerability	Detained/Held	Health Problems	Child at Risk	Woman at Risk	Other	*p*-Value *	Cramer’s V
n	451	150	79	62	64	96	-	-
Age (years), mean (SD, min–max)	20.3 ± 8.9 (0–63)	19.6 ± 7.0 (0–36)	24.8 ± 8.6 (0–57)	8.0 ± 6.6 (0–17)	22.9 ± 5.0 (17–44)	23.8 ± 8.1 (0–63)	-	-
Signs, skin manifestations or symptoms of contagious infectious conditions							0.018923	-
None, n (%)	363 (80.5%)	110 (73.3%)	63 (79.7%)	49 (79.0%)	58 (90.6%)	83 (86.5%)		
Present, n (%)	88 (19.5%)	40 (26.7%)	16 (20.3%)	13 (21.0%)	6 (9.4%)	13 (13.5%)		
Other health findings							**<0.000001**	0.1815022
None, n (%)	281 (62.3%)	107 (71.3%)	45 (57.0%)	45 (72.6%)	46 (71.9%)	38 (39.6%)		
Present, n (%)	170 (37.7%)	43 (28.7%)	34 (43.0%)	17 (27.4%)	18 (28.1%)	58 (60.4%)		

* Chi Square Test. **Bold:** *p*-value less than 0.01.

**Table 9:** Results of inferential analysis by level of education.

	None or Informal	Between 1 and 5 Years	Between 6 and 8 Years	Equal or Higher Than 9 Years	Unknown	*p*-Value *	Cramer’s V
n	216	61	90	72	811	-	-
Age (years), mean (SD, min–max)	12.1 ± 11.5 (0–55)	20.5 ± 6.7 (7–42)	21.6 ± 7.1 (13–48)	24.0 ± 6.9 (12–46)	24.2 ± 7.9 (0–67)	-	-
Signs, skin manifestations or symptoms of contagious infectious conditions						0.176687	-
None, n (%)	166 (76.9%)	51 (83.6%)	71 (78.9%)	60 (83.3%)	679 (83.7%)		
Present, n (%)	50 (23.1%)	10 (16.4%)	19 (21.1%)	12 (16.7%)	132 (16.3%)		
Other health findings						0.827120	-
None, n (%)	152 (70.4%)	40 (65.6%)	59 (65.6%)	47 (65.3%)	538 (66.3%)		
Present, n (%)	64 (29.6%)	21 (34.4%)	31 (34.4%)	25 (34.7%)	273 (33.7%)		

* Chi Square Test.

## Data Availability

The raw data supporting the conclusions of this article will be made available by the authors on request.

## Abbreviations

The following abbreviations are used in this manuscript:

EU	European Union
ES	Effect Size
FCEI	Italian Federation of Evangelic Churches
HBV	Hepatitis B Virus
HCV	Hepatitis C Virus
HIV	Human Immunodeficiency Virus
INMP	Istituto Nazionale per la promozione della salute delle popolazioni Migranti e per il contrasto delle malattie della Povertà
NCDs	Non-Communicable Diseases
NIHMP	National Institute for Health, Migration and Poverty
PEPs	Protected Entry Procedures
SSN	Servizio Sanitario Nazionale
TB	Tuberculosis
UFM	Unaccompanied Foreign Minor
UNHCR	United Nations High Commissioner for Refugees
